# Clinical guidelines for complex extremity war wound management: update and consensus using a mixed-method approach

**DOI:** 10.1093/bjsopen/zraf173

**Published:** 2026-03-05

**Authors:** Samuel Snelling, Henry Claireaux, Harrison Roocroft, Kyung-Hoon Moon, Johann Jeevaratnam, Neil Eisenstein, Robert M T Staruch

**Affiliations:** Department of Research & Clinical Innovation, Academic Department of Military Surgery & Trauma, Birmingham, UK; Department of Research & Clinical Innovation, Academic Department of Military Trauma & Orthopaedics, Birmingham, UK; Department of Materials, Imperial College London, London, UK; Centre for Bacterial Resistance Biology, Imperial College London, London, UK; Department of Research & Clinical Innovation, Academic Department of Military Surgery & Trauma, Birmingham, UK; Department of Research & Clinical Innovation, Academic Department of Military Trauma & Orthopaedics, Birmingham, UK; Centre for Injury Studies, Imperial College, London, UK; Joint Hospital Group, Defence Medical Services, UK; Department of Research & Clinical Innovation, Academic Department of Military Trauma & Orthopaedics, Birmingham, UK; Department of Research & Clinical Innovation, Academic Department of Military Surgery & Trauma, Birmingham, UK; Department of Plastic & Reconstructive Surgery, Oxford University Hospitals NHS Trust, Oxford, UK

## Abstract

**Background:**

Extremity trauma is a common and significant injury sustained by military and civilian casualties of war. Civilian management has evolved, adopting a multidisciplinary orthoplastics approach. Accurate and timely management of open fractures and complex war wounds is required to minimize complications and optimize outcomes. The Lower Limb Debridement for Operations Working Group is part of the UK Defence Medical Services and aimed to provide updated guidelines to support deployed surgeons, given the modern nature of conflict.

**Methods:**

The working group formed a panel of military consultants (attendings) in Trauma and Orthopaedics and Plastics and Reconstructive Surgery. The literature was systematically reviewed for new evidence. A modified Delphi technique was adopted, and an initial survey was circulated to the working group to gain its opinion on current guidance. Responses were used by the steering group chairs to formulate updated guidance on combat wound management. A consensus meeting with consultants (attendings) was then used to agree the final guidance.

**Results:**

Eight previous recommendations were removed and 21 new recommendations were formed, providing updated guidelines. Recommendations relate to timing, location, and technique of wound excision including irrigation and requirements for wound closure.

**Conclusions:**

Civilian and military combat casualties require well prepared surgeons and evidence-based guidance to save life and limb. These recommendations represent a consensus, utilizing up-to-date literature and expert opinions of both orthopaedic and plastic surgeons. In large-scale combat operations, NHS surgeons working in the UK may be required to treat large numbers of patients repatriated from conflict. These guidelines may form a useful part of their preparation.

## Teaser

Extremity trauma is a major injury in military and civilian conflict, requiring accurate and timely multidisciplinary management to optimize outcomes. The UK Defence Medical Services convened a panel of consultant orthopaedic and plastic surgeons, who conducted a systematic review and Delphi consensus process to update guidance on combat wound management. The final recommendations provide evidence-based support for deployed and NHS surgeons who may be likely to treat repatriated casualties in a large-scale combat operations.

## Introduction

Extremity trauma is a common and significant injury in contemporary conflict, sustained by service persons and civilians alike^1–8^. Accurate and timely multidisciplinary management of open fractures and complex war wounds is required to minimize the risk of complications and optimize patient outcomes^1,9–11^. Whilst previous military doctrine centred around counterinsurgency or contingency operations, current planning is towards peer-to-peer or large-scale combat operations (LSCOs), hitherto not seen since the Second World War.

Peer-to-peer conflict refers to war between technologically advanced nations with comparable military capabilities^12^. LCSOs are those that are intensely lethal and destructive involving multiple military divisions characterized by widespread violence across all domains (land, air, sea, space, and cyber). LSCOs impose immense strain on national resources, leading to high casualties with enormous logistical burdens and resources^13,14^.

The UK Defence Medical Services (DMS) have developed clear approaches to moving casualties from the point of wounding through a casualty chain to forward surgical facilities where damage control surgery can take place. Furthermore, this evacuation chain can deliver treatment to patients whilst in transit. Patients move through this pathway to rearward surgical facilities for further surgery and then definitive reconstruction. This process is called the operational patient care pathway (OPCP)^15^, and is explained in more detail below. Role 1 provides deployed primary care and initial pre-hospital care; Role 2 is the most forward surgical/hospital capability; Role 3 is the rearward deployed surgical/hospital capability; and Role 4 represents the UK-based Royal Centre for Defence Medicine (RCDM). The RCDM is collocated within the Queen Elizabeth Hospital, Birmingham, an NHS facility within the University Hospitals Birmingham NHS trust. Currently there are no dedicated military hospitals in the UK, as these were closed as a result of the 1994 paper *Front Line First: The Defence Costs* study^16^. If the UK were to be engaged in LSCOs, medical care of combat casualties may need to expand outside of the RCDM and involve other NHS facilities in a co-ordinated fashion. A high proportion of regular and reserve military clinicians would likely be called back into active service in order to deploy or prepare to deploy. Therefore, NHS surgeons may be required to step into roles co-ordinating and treating large numbers of patients repatriated from conflict as they are distributed around the healthcare system, as was seen during previous conflicts.

The asymmetrical, counterinsurgency operations experienced by UK Armed Forces in Iraq and Afghanistan (Op TELIC and HERRICK), led to the development of a well established and well organised OPCP^17^, enabling the highest standards of care to be delivered from the point of wounding to definitive Role 4 care in the UK. Towards the end of the conflict, survival rates following trauma were increasing due to standardized training on pre-hospital haemorrhage control, physician-led retrieval teams (medical emergency response teams), and early resuscitation with blood products^18^. Air superiority allowed MEDEVAC (medical evacuation) to move casualties rapidly from the point of wounding to Role 3 for damage control surgery and then onward to Role 4 for definitive reconstruction, often being treated at the RCDM within 48 hours (h) of injury^19^.

At the time of writing this article, the ongoing conflict in Ukraine differs significantly from the NATO experience in Afghanistan and Iraq^7,8^. Increased lethality and volume of munitions have led to larger numbers of more severely injured casualties. Seventy per cent of Ukrainian casualties are from rocket attacks or artillery. Lack of air superiority as well as targeting of medical assets and MEDEVAC mean severely injured casualties are held for significant periods of time, placing strain on forward surgical facilities and increasing time from wounding to definitive care. Limb injuries form the majority of the surgical workload^7^. Large numbers of patients with wounds, including open fractures, infected with extensively drug-resistant bacteria have been identified^20^. It may be that patients with life-threatening haemorrhage do not survive to medical facilities in this operating environment. Therefore, work limiting morbidity and mortality from infection should be optimized.

In 2011, the UK DMS generated 29 recommendations for management of complex limb injury^1^ (*Table 1*). Since this paper was published, there has been further development of civilian and military experience. In 2020, in the civilian setting, the British Orthopaedic Association (BOA) and British Association of Plastic, Reconstructive and Aesthetic Surgeons (BAPRAS) produced the most recent Standards for the Management of Open Fractures^9,21^.

**Table 1:** Previous 2011 recommendations, including outcomes

	2011 Recommendation	Outcome in 2025 Version
1	Debridement of limb wounds should be undertaken as soon as practical	Updated
2	Debridement of limb wounds should be undertaken in an operating theatre	Updated
3	Preoperative washing with copious volumes of warmed detergent solution is essential	Updated
4	Pre- and post-debridement photography is an essential part of the patient record	Updated
5	Macroscopically non-viable tissue should be excised	Updated
6	Traumatised but potentially viable tissue may be left if serial debridement is guaranteed	Updated
7	It is not necessary to excise the skin edges in the absence of macroscopic injury	
8	Undermining skin by over generous excision of fat/fascia is to be avoided	Removed
9	Fasciotomy should be along the full length of the muscle compartment	Updated
10	Adequate skin bridge should be preserved between fasciotomy incisions and between traumatic wounds and fasciotomy incisions	Removed
11	Wound extension dissection should be in the subfascial plane	Updated
12	The use of diathermy is not recommended other than for control of bleeding	Removed
13	Non-contractile muscle of altered consistence should be excised	Removed
14	Formal muscle flaps should not be fashioned at the initial debridement	Updated
15	Suture tagging of divided nerve and tendons should not be performed	Removed
16	The status of vital structures encountered during debridement should be carefully documented	Updated
17	It is not necessary to locate neurovascular structures solely in order to document their status	Updated
18	Primary reconstruction of tendons and nerves should not be performed at the initial debridement	Updated
19	Contaminated bone fragments with a very tenuous or no soft tissue attachment should be excised	Updated
20	Irrigation should be performed with warmed sterile physiological saline where available	Updated
21	Irrigation should be performed using a low-pressure delivery system such as a syringe, giving set or cutting the corner off a fluid bag	Updated
22	9 L of irrigation should be used for complex blast wounds, 6 L for penetrating ballistic injury, and 3 L for superficial wounds	Removed
23	Dressing should be well secured to prevent displacement during evacuation	Updated
24	Traditional sterile gauze and topical negative pressure are both acceptable wound dressings	Updated
25	Where the surgeon is entirely happy with the debridement delayed primary closure should be performed at around 5 days	Updated
26	Where traumatised but potentially viable tissue has been left, the wound should be inspected in the operating theatre at around 48 h	Removed
27	In the context of complex blast, explosion wounds initial debridement should be undertaken whenever the opportunity permits	Updated
28	A philosophy of serial marginal debridement is indicated for the majority of complex blast/explosion wounds	Removed
29	Some low-energy narrow channel and superficial soft-tissue wounds do not require full surgical debridement	Updated

L, litres; h, hours.

## Military contextual factors for the civilian audience

For the benefit of civilian surgeons reading this paper, some additional explanations have been added to the doctrinal context of the recommendations, to help understanding relating to the non-clinical and logistical aspects of conflict causality care.

**Fig. 1:**
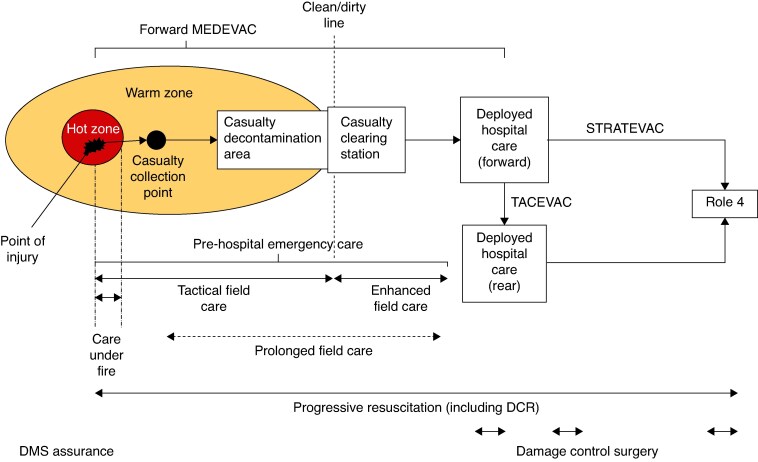
OPCP diagram outlines the patient evacuation pathway for injured casualties OPCP, Operational Patient Care Pathway; MEDEVAC, medical evacuation; STRATEVAC, strategic evacuation; TACEVAC, tactical evacuation; DCR, damage control resuscitation; DMS, Defence Medical Services.

The overarching process in the transport and treatment of any casualty is termed the ‘Operational Patient Care Pathway (OPCP)’ (*Fig. 1* Crown Copyright, reproduced under the CC BY 4.0 International License). This is set out in NATO Doctrine: Allied Joint Publication 4.10^22^.

The UK Military follows prescribed timelines of providing certain levels of care at specific time points; this is termed 10.1.2(2) + 2. The ‘Hot Zone’ is an area of operation where active combat is ongoing. All military personnel are trained in basic first aid and within 10 minutes (min) of injury a casualty will receive ‘care under fire’, consisting of the application of tourniquets and basic supportive airway manoeuvres. The casualty is then evacuated to reach a ‘casualty clearing station’, also termed Role 1, within 1 h. In Role 1, specifically trained and qualified medical personnel can deliver advanced resuscitation and pre-hospital emergency care. Following further evacuation, the casualty should arrive to deployed hospital care forward, also termed Role 2, and undergo damage control resuscitation and damage control surgery; this is expected to be within 2 h of injury and take no longer than 2 h. Deployed hospital care (rear), also termed Role 3, is an in-country hospital facility with more specialist medical and diagnostic capability. Any additional surgical or diagnostic intervention required to necessitate safe evacuation out of the country needs to be completed within a further 2 h. TACEVAC refers to tactical evacuation, an evacuation that takes place in synergy with an ongoing combat operation using an appropriate evacuation frame (air, land, sea) to another deployed facility. STRATEVAC refers to strategic evacuation, an evacuation that takes place from the rearmost deployed hospital facility back to the UK or ‘firm base’ facility (Role 4) for ongoing treatment^15^.

## Methods

### Study design and ethics

This study was registered with the Academic Department of Military Surgery and Trauma (ADMST). The project was led and directed by the steering committee. As there was no direct patient involvement, approval from the Ministry of Defence Ethics Committee was not sought. Public or patient involvement was not sought.

**Fig. 2:**
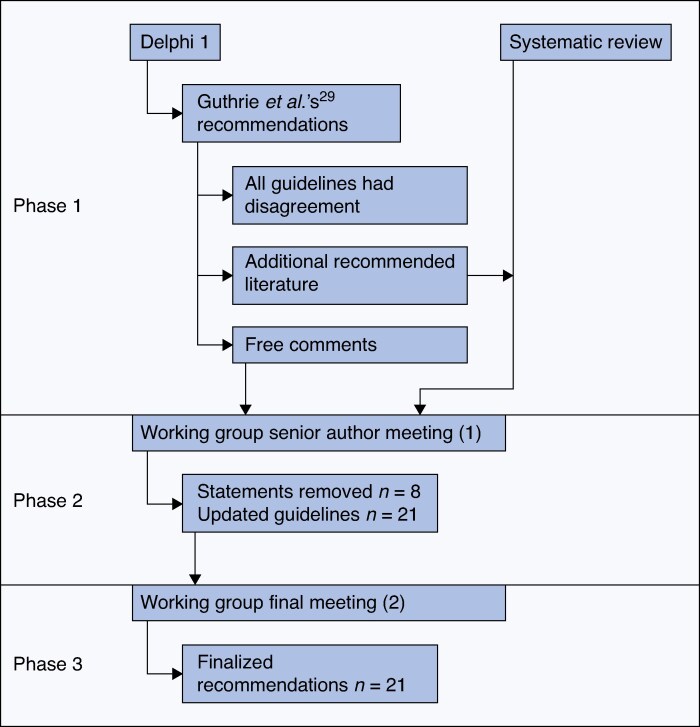
Outline of methodology

A mixed methodology involving a literature review and modified Delphi approach were used to generate consensus for the new clinical guidelines; see *Fig. 2*.

The AGREE checklist for describing the practice guidelines^23^ and the ACCORD checklist for Consensus statements^24^ were followed.

### Evidence review

Between 3 July and 18 July 2024, a preparatory literature review was completed. MEDLINE, Google Scholar, and PubMed were utilized to search for keywords. Citations for each original recommendation were reviewed and cross-referenced with evidence from the most recent BOA/BAPRAS guidance. Bibliographic review on subsequent evidence was completed. Where applicable, new evidence was sought from subject matter experts and mapped to recommendations where appropriate. There was no date limit for evidence included. Reviews were conducted in line with James Lind Alliance methodology^25,26^.

### Steering group and nominal group panel composition

A steering group of two senior consultants (attendings), one in Trauma and Orthopaedics and one in Plastics and Reconstructive Surgery, alongside a postdoctoral academic surgeon, formed a three-person steering committee to oversee the process. The working group panel consisted of consultants (attendings) in Trauma and Orthopaedics and Plastics and Reconstructive Surgery with subspeciality interest in extremity reconstruction and experience in war surgery. The total number of panel members was 13, with 12 being male and 1 female surgeon. All panel members engaged in each phase of consensus review.

### Round 1 questionnaire

Between July and August 2024, a first-round questionnaire was adopted to survey the panel for their opinions on the current guidelines and their applicability to the modern conflict environment.

The questionnaires outlined Guthrie *et al.*’s original recommendations along with matched references from the original paper. The questionnaire was formed of four questions for each of the 29 recommendations, as well as free-text responses. The questions are outlined in *Table 2*.

**Table 2:** First-round questionnaire to panel members

	Question
1.	Do you agree this recommendation should continue it its current form?
2.	If you believe this recommendation should not continue in its current form, please explain why?
3.	How would you change this recommendation?
4.	Please supply evidence (up-to-date literature evidence) to support your recommendations.

Consultants answered each of the four questions for each of the 29 recommendations.

### Steering group consensus meeting

The steering group met to analyse the first-round questionnaire responses and generate a new draft set of recommendations.

### Working group final meeting

The panel had a final consensus meeting where all new recommendations were discussed in detail alongside all available evidence. A vote was held at the consensus meeting and, where unanimity was not reached, further structured discussion took place until the panel arrived at each recommendation.

### External review

Defence Professor of Surgery, ADMST, was invited to externally review this paper to ensure quality and feedback on draft recommendations.

#### Limitations and scope of these guidelines

Priorities of treatment for military wounded are to preserve life with haemorrhage control and prevention of infection, followed eventually by reconstruction; allowing the injured back into the fight as well as maximizing function following service^21^. Increasingly, civilian surgeons are deploying to conflicts with humanitarian organizations to support and treat casualties, and the hope is that they can reference these guidelines to aid in their surgical management of war wounds. These guidelines apply to the management of combat injured patients within Role 2 and Role 3 settings only (deployed surgical care) for the DMS. Management of combat wounds before this is beyond the scope of these guidelines and will be referenced in a separate guideline. Management of these wounds at Role 4 should adhere to UK standards of practice. Additionally, not all wounds encountered in the deployed setting require surgical care. This guidance applies only to those patients who require surgical management of their wounds as judged by the deployed team. These guidelines do not cover the management of more complex war wounds, specifically those with structural damage to the head and neck, perineum, and abdomen. More detailed guidance on how to manage these wounds in the acute setting will follow.

These guidelines have been produced to provide a handrail for the deployed surgeon, whether military or civilian. These guidelines are to be interpreted alongside predeployment education and training, and are not designed to be an exhaustive manual of how to perform wound excision. Contextual factors are fundamental to decision-making in the deployed setting. These guidelines have been written with the intention of providing helpful direction while, at all times, emphasizing the importance of surgical judgement. Additionally, these guidelines may form a useful part of the preparation for NHS surgeons who may be called upon to treat combat casualties repatriated to the UK and distributed around the health system. Deviation from these consensus statements may be justified by a wide range of contextual factors that include but are not limited to the tactical situation; timeliness and reliability of patient movement into and out of the facility; equipment and resource supply levels; and volume of cases.

## Results

### Literature review and mapping

Thirty-seven separate research articles or publications were mapped to the new recommendations.

### Clinical guidelines: combat wound excision summary recommendations

**1. Patients with combat wounds should undergo wound excision within the following timelines:**
  - As soon as possible for heavily contaminated wounds.
  - Within 12 h for high-energy-transfer injuries.
  - Within 24 h for all other wounds requiring surgery.

What was previously referred to as ‘debridement’ is now described as ‘wound excision’, to match National Institute of Clinical Excellence (NICE)^27^ and BOA/BAPRAS^9^ guidance. This change reflects the aim to encourage the surgical conversion of a complex war wound to a clean surgical wound with the excision of non-viable tissue rather than just dilution of contamination.

Operational factors will directly affect the layout, footprint, and location of surgical capabilities in each area of operations. Similarly, timing to surgical excision will be dictated by many factors. The detail of how each of these factors may affect the OPCP and subsequently timing and location of wound excision is outside the scope of these recommendations. However, NATO planning guidelines and JSP Health Service Support to Land Operations provide overarching doctrinal support^22^. The OPCP should aim to deliver patients to surgical facilities within the timelines of 10.1.2(2) + 2.

Despite operational considerations for timing, certain clinical actualities remain. Heavily contaminated wounds are at significant risk of infection and require earlier wound excision to prevent further bacterial or fungal proliferation and subsequent deep infection^28–30^. The recommendations are that heavily contaminated wounds (for example, blast or contamination with sewage, seawater, etc.) require immediate wound excision, high-energy wounds (for example gunshot or shrapnel wounds without gross contamination) require wound excision within 12 h^21,31^, and all other wounds require wound excision within 24 h^9,10,32^.

**2. Wound excision should be undertaken in a surgical facility.**

A ‘surgical facility’ is defined here as wherever the deployed surgical team has designated as its ‘operating theatre’. This may be in a tented structure, aboard ship, on a suitable vehicle, in a building of opportunity, or any other designated location. Formal surgical wound excision should not take place forward of Role 2. For Role 1 management of complex wounds, the specific Clinical Guideline for Operations (CGO) should be followed.

**3. Gross contamination should be removed before surgery by irrigation. Potable water is acceptable for this purpose.**

Use enough fluid to remove all loose contamination^33–36^. Be as thorough as resources allow. Some contamination will inevitably remain embedded in tissues and will require surgical removal.

**4. Pre- and post-excision wound photography is strongly encouraged as part of the patient record.**

Access to ElectroMed and operational personal electronic device policies may be limiting factors but medical photography of the injuries is best practice and allows for accurate surgical planning^9,37^. Seek guidance from the chain of command regarding the mechanism for capturing, storing, and transmitting these images. Every effort should be made by the deployed command team to facilitate this process.

**5. Wound excision should be performed to remove all non-viable tissue. Nerves and blood vessels should be preserved wherever possible.**

A systematic and meticulous approach should be used when undertaking wound excision^9,38^. The aim is to remove all contamination and non-viable tissue^39,40^. This is not a quick procedure and the consequences on the physiology of the patient and theatre utilization must be accounted for.

The surgeon should proceed from superficial to deep, ensuring that all contamination and non-viable tissue is excised at each level: skin, subcutaneous fat, fascia, muscle, and bone. A ‘clockface’ approach is recommended at each level, whereby the surgeon works from ‘12 o’clock’ around the wound to ensure comprehensive assessment.

If part or all of a limb is clearly beyond salvage, given the contextual factors, it must be debrided in the same manner as any other non-viable tissue. It is not considered an ‘amputation’ in the same manner as those performed in firm base practice. See Consensus Statement 11 below.

Surgeon judgement is required to assess what is and is not viable tissue. Where there is doubt about the viability of tissue there should be an assumption that excision is safer than retention; see Statement 7 for additional contextual detail. However, nerves and blood vessels are a special case and should be preserved unless they are clearly beyond salvage.

Limb-threatening vascular injury is outside the scope of this guideline and is covered in a separate CGO. Always determine whether a vessel is the main supply to the distal limb before ligation. If bleeding, non-named vessels that are not essential for limb or muscle compartment perfusion can be clamped/tied to prevent further blood loss. If in doubt, apply a temporary clamp to the vessel in question to gain proximal control, and then reassess distal pulses to evaluate extremity blood flow.

**6. At the primary excision, non-viable skin at the margins of the wound should be carefully excised to leave edges that bleed from the dermis. This does not need to be repeated during subsequent wound excision if the skin edges remain healthy** ^9,41^.

Military wounds are dynamic and can change over time as the effect of blast or ballistic trauma can have delayed effects on the tissues; this phenomenon is called progressive tissue necrosis^19^. Wound assessment should take place at each surgical stage. Further excision is only necessary if previously viable tissue has advanced to become non-viable. Over-excision during subsequent surgery will result in unnecessary removal of tissue. See recommendation 20 for requirements for wound closure or reconstruction.

**7. In a mature OPCP, serial marginal wound excision may be an appropriate strategy to avoid excessive removal of viable tissue in evolving complex wounds.**

This consensus statement reflects the fundamental importance of contextual factors in how combat wounds are managed and is a modification of Consensus Statement 5. In very specific deployed settings where resources are plentiful relative to patient volume, the surgical decision-making may permit the retention of tissue with intermediate viability on the assumption that the patient will be able to return to theatre for further review and removal of tissue that is non-viable^1,9,19,32^. Where early return to theatre for serial excisions cannot be guaranteed, excision is safer than retention.

**8. If acute compartment syndrome is suspected, this should be managed according to the Acute Compartment Syndrome & Fasciotomy CGO** ^42–45^.
**9. Where necessary, wound extensions should be along fasciotomy lines if possible.**

Wound extensions, and their length, are determined by the size and shape of the wound as well as the need to access deeper structures. The surgeon must access the full extent of the wound cavity and explore tissue planes along which contamination can spread^9^. If the wound is complicated by a fracture, wound extensions are required to allow full delivery of both ends of the bone out of the wound for irrigation and removal of contamination.

**10. Skin degloving should be avoided. Wound extension should be in the subfascial plane.**

Do not undermine skin.

**11. Flaps should not be fashioned during primary excision, and all viable tissue should be kept.**

Anatomical flaps such as those fashioned for closing amputation residua are not indicated in the deployed setting. All viable tissue, regardless of anatomical pattern, should be retained. This maximizes the reconstructive options available upon evacuation to definitive care.

**12. Suture tagging of divided nerve and tendons is not required.**
**13. The status of neurovascular structures encountered during wound excision should be carefully documented** ^9,37,46^.
**14. It is not necessary to locate neurovascular structures solely in order to document their status.**

The factors that determine the extent of the surgical extension are discussed in Consensus Statement 9. Neurovascular structures that lie outside the required surgical field should not be exposed unnecessarily^9,47^.

**15. Primary repair/reconstruction of tendons and nerves must not be performed at the primary excision. Definitive repair should be considered when the wound is undergoing delayed primary closure at a subsequent episode.**

Nerves and tendon injuries may be reconstructed at a later date, if necessary. They are of secondary importance to achieving a healed wound and should not dictate the manner and timing of wound closure. Note that the word ‘considered’ does not indicate any degree of compulsion but permits an attempt at repair if the time, resources, equipment, and skill set are available.

**16. Bone fragments with a tenuous or no soft tissue attachment should be excised.**

Orthoplastic reconstruction using mechanically relevant devitalized bone (ORDB) is a recognized technique in civilian practice^48,49^. However, there is no published evidence of its use in the military population and it is likely not applicable to deployed wound excision due to lack of technical means of securing bone fragments; poor sterility of initial surgical facilities in comparison with civilian operating theatres; the high nature energy of war wounds with progression over time and potential for serial excisions; and the delay to definitive reconstruction. Given these factors, there is no current role for the retention of devitalized bone fragments, no matter how orthopaedically important, in the deployed setting.

**17. Intraoperative irrigation of contaminated wounds should be performed using copious low-pressure sterile saline where resources allow. If sterile saline is unavailable, potable water may be used as an alternative** ^9,27,31,33–36,50–52^.

Following excision, the wound should be copiously washed to attempt to remove any remaining bacteria. Use enough irrigation to achieve a surgically clean wound. Where resources allow, a minimum of 3 litres (L) is recommended. Significantly higher volumes may be required in complex wounds. For the irrigation of open fractures, the FLOW trial demonstrated no statistical difference in reoperation rates between low pressure and high pressure and found normal saline to be preferrable to soap solution^34,35^. Normal saline is regularly held in Role 2 modules and therefore should be used. If it is not available due to supply issues, systematic reviews (including a Cochrane review) have shown no increased rate of infection or effect on wound healing using tap water *versus* normal saline, and therefore potable water may be used as an alternative^33,53^.

**18. High-pressure or pulsed lavage should not be used in the management of combat wounds.**

High-pressure lavage has been demonstrated to drive bacteria deeper into tissues, strip periosteum, and cause tissue injury^54–57^. Furthermore, it requires additional equipment not found in surgical modules.

**19. Following initial wound excision, simple dressings should be used as follows:**
  - Non-adherent layer in direct contact with the wound bed and edges.
  - Either an adhesive outer dressing or gauze/wool/crepe depending on size and location. This must be well secured.

Where available, negative pressure wound therapy (NPWT) systems may be used to manage complex and/or high-exudate wounds^58–61^; their availability may be limited in the deployed environment. A Cochrane review^61^ showed no benefit for NPWT *versus* conventional dressings, and therefore deployed surgeons can confidently utilize conventional dressings as described above. Use a non-adherent interface between the wound bed and secondary absorbent gauze to prevent adherence that can cause pain and further damage the wound bed at the next change of dressing.

Do not underestimate the tendency for dressings to become displaced during patient evacuation^1^. This can have significant negative effects for the patient and transport assets. Secure all dressings to withstand robust handling.

**20. Surgeons should perform delayed primary closure at 3–5 days if the following conditions are met** ^9,10,38,62^:
  - No evidence of infection.
  - Tissues are healthy, with bleeding edges.
  - No evidence of deterioration since last excision surgery (no new necrotic or infected tissue).
  - The wound can be closed without excessive tension.

Wounds should ideally be reassessed at 24–48 h to assess for the above. At each stage the wound should be irrigated and new dressings applied. Guidance on types of dressings will be available in wound management guidance (in development). If the above conditions are not met, the patient requires re-excision surgery. Ensure that record-keeping and photography, as described above, are undertaken at each surgical event^37^.

Reconstructive options when the above criteria are met is outside the scope of this guideline.

**21. Some low-energy-transfer, narrow channel, and superficial wounds do not require full surgical excision** ^21^.

Refer to the CGO Gunshot Injuries to Extremities for further guidance^63^.

All CGOs referenced in the guideline are updated at regular intervals. Online versions should be sought for the most up-to-date guidance^64^.

## Discussion

The nature of conflict has evolved over the past decade and, as such, so has the way that medical care must be provided. Hippocrates once said to become a surgeon ‘*first they must go to war*’, underscoring a long-held truism that what is learnt in battlefield medicine often trickles down into civilian practice. However, in this work the reverse is demonstrated as evidence for delivering ‘gold standard’ civilian care is translated for use on the modern battlefield. Specifically, the evolution in orthoplastic care of open-extremity fractures has translated into the increasing deployment of plastic surgeons in forward surgical teams. This fact is reflected in the interdisciplinary working of these two specialities within the DMS. The BOA/BAPRAS civilian guidance is largely focused for use by fellowship-trained surgeons in extremity reconstruction, or the general consultants in Trauma and Orthopaedics or Plastics and Reconstructive Surgery in a UK district general hospital. In contrast, the military clinical guidelines for operations are aimed at the deployed military surgeon, who may come from a variety of subspeciality backgrounds.

Prolonged casualty evacuation timelines are expected in future conflict. In Afghanistan, a casualty could be treated in the pre-hospital environment, be air evacuated back to the hospital at Camp Bastion, and be on an operating table receiving damage control surgery within one hour^17^. Whereas, in future operations, forward facilities may be expected to treat casualties for several hours, or even days, until evacuation is possible. These operational constraints necessitate a degree of divergence from the BOA/BAPRAS standards. As such, these guidelines advise that wound excision should take place as soon as reasonably possible. This may mean this operation takes place later than in civilian practice. Furthermore, the operational situation may limit the volumes of sterile saline available for wound washouts. Therefore, clean potable water may be used to wash out wounds and reduce contamination. This guideline does not define what constitutes a surgical facility and it is appreciated that the operational situation, number of casualties, and medical staff available will vary significantly.

Pre- and postoperative photography of wounds is important for surgical planning and documentation. Telemedicine reach-back systems, such as the mobile app Pando (Forward Clinical Ltd, London, UK), provide a secure link between deployed clinicians and secondary-care specialists via a smartphone for advice, including review of images^65^. This system has been in use by the UK DMS since 2019, though the contracted supplier may change in the future^66^. However, such systems emit an electronic signature that may enable the enemy to identify a medical facility. In conflict against a peer or near-peer adversary, such systems may be denied due to the risk of attack, such as with drones or artillery. This guideline, therefore, recommends that wound photography should be performed where feasible.

In the future operating environment, casualty volumes may overwhelm the medical infrastructure deployed to support them. A broad spectrum of wounds will be seen by clinicians, and this guidance supports the use of clinical judgement on which require surgical debridement. Such judgement will be invaluable in ensuring that facilities are not overwhelmed or exhausted of consumables too quickly.

Concerning the surgical technique for wound excision, this guidance aims to provide straightforward concepts to guide the military surgeon. Unlike in the civilian clinical setting, both timelines for patient evacuation and capability of deployed surgical facilities are fluid and potentially unpredictable. As such, clinicians cannot assume that they themselves will be performing any subsequent wound excision, nor when any subsequent surgery may take place. Therefore, primary excisions should be as definitive as possible, aiming to provide a clean wound with bleeding skin edges, which is extended along fasciotomy planes with careful documentation of encountered anatomical structures. ORDB has been demonstrated in civilian cohort studies^48,49^ to lead to equivalent health-related quality-of-life outcomes as control groups. However, the following factors mean that retention of ORDB in the deployed setting is not recommended: the severity and nature of combat wounds, degree of contamination of the wounds, the prevalence of antibiotic-resistant infection in this group, the challenges of sterility in the deployed setting, and the lack of surgical equipment required to stabilize such fragments.

The original guidance outlined specific volumes of fluid for the irrigation of increasingly complex combat wounds. It was postulated that a minimum of 9 L of sterile saline should be used for complex blast injuries, 6 L for penetrating ballistic injury, and 3 L for superficial wounds. In the updated guidelines, specific volumes have been omitted. Conflict wounds are highly contaminated and require extensive wound excision and lavage. However, the volume of fluid used could be limited by available resources. Constraining surgeons to a specific volume, especially large volumes of sterile fluid, will create significant logistical burdens with the anticipated large number of casualties associated with LSCOs. Given that the authors found no evidence for these prescribed volumes, this constraint was removed. Surgeons should aim to achieve a macroscopically clean wound with the resources available, rather than be constrained by arbitrary fluid volumes. Finally, although evidence does support the use of large-volume sterile saline lavage to reduce contamination, the previously postulated specific volumes for blast, penetrating, and superficial wounds were not evidence-based.

The WOLLF trial^60^ demonstrated that topical negative pressure is equivalent, but not superior, to sterile soaked saline gauze in the management of incisional wounds. The degree of tissue trauma, contamination, and infection risk from a blast or ballistic injury is far higher than with an incisional wound. Clinical experience from operations in Afghanistan demonstrated the utility of negative-pressure dressings in reducing odour and robustness in transport. Whilst not yet proven through quantitative research or patient-reported outcome measures, these are two important factors in military care that cannot be underestimated. Patients may be transported large distances over many days in a variety of platforms (road, rail, air) without undergoing further dressing changes. Therefore, any dressing must be capable of remaining intact and providing adequate control of wound exudate and odour. Whilst widespread use of negative-pressure therapy may be perceived as advantageous, this may not be feasible due to constraints on large-scale manufacturing and logistical constraints across the operational care pathway. Hence, the use of standard dressings (soft wool and crepe) alongside an effective non-adherent interface should be both an accepted and well rehearsed standard of practice amongst surgeons. In particular, the use of an effective interface layer against the wound is critical in reducing iatrogenic injury to key anatomical structures.

The guidelines do not discuss the use of antibiotics. Numerous large case series data^67–69^ from the United States experience in Iraq and Afghanistan, as well as other conflict zones, have outlined how these wounds are heavily contaminated with multiple organisms (both bacteria and fungi). Furthermore, evidence^70,71^ from the conflict in Ukraine has highlighted the increasing incidence of wound infection with multidrug-resistant bacterial strains and fungal organisms that warrant the additional use of antifungal therapy. This further complicates the antimicrobial management of these patients. In the deployed setting, current guidance advises the early use of antibiotics (ideally within 1 h of injury) by forward-deployed clinical teams. In future conflict environments, the use of antibiotics as early as feasibly possible and at regular intervals until definitive surgical excision would be advised. This is laid out in the deployed antimicrobial guidance document^72^. This document can be changed in real time in response to military medical intelligence and thus make any recommendation in this guidance outdated. Civilian colleagues should be cognisant of the level of severity of the wound encountered from combat, and their association with deep-seated macro- and microscopic contamination that necessitates early microbiology input and antimicrobial and antifungal therapy. Again, in an LSCO, the time for casualties to reach a definitive surgical facility, the availability of antibiotics, and the type of available antibiotic may all influence the capacity of clinicians to achieve ‘gold standard’ care. Instead ‘best effort’ care may be all that is available or sufficient to achieve the greatest good for the greatest number. These are important considerations for civilian clinicians in these scenarios whose only comparable situation was the COVID-19 global pandemic.

High-quality interventional research is primarily conducted in the civilian setting for ethical and operational reasons. As a result, the vast the majority of military research lacks randomization. Therefore, these guidelines balance extrapolation of Level 1 evidence from methodologically robust civilian research alongside military observational research by utilizing a consensus approach to the opinions of experts with significant operational experience.

This work adapted well published techniques of consensus methodology, specifically a modified Delphi technique that has been well developed within healthcare research. The strict methods of a Delphi approach, utilizing multiple rounds of voting and panel discussion to achieve consensus, were avoided. Panel members were blinded to the responses of other members after the first-round questionnaire. The steering group then used these to generate consensus or understand areas for discussion and therefore guide the second-round panel discussion. This work did not use a formal second questionnaire for voting on consensus statements but instead adopted voting during the online meeting to achieve consensus. This represents the first time that such a technique has been used to develop clinical guidelines for military operations. The authors propose that this rigorous and evidence-based approach be adopted more widely within the DMS for the development of updated clinical guidelines.

These guidelines provide both a comprehensive and pragmatic handrail for the deployed military surgeon in the management of combat wounds requiring surgical excision. They may form a useful part of the preparation for NHS surgeons called upon to treat large numbers of combat casualties distributed throughout the healthcare system. This guidance has aimed to ‘translate’ the ‘gold standard’ guidance provided by BOA/BAPRAS for lower-extremity open fractures for future conflict environments.

These recommendations represent a consensus, utilizing up-to-date literature and expert opinion of consultants (attendings) both in Trauma and Orthopaedics and Plastics and Reconstructive Surgery, which provide useful guidance for civilian and military surgeons in a variety of complex situations.

## Collaborators

Members of the Lower Limb Debridement for Operations Working Group: Wg Cdr George Wheble (North Bristol NHS Trust, Bristol, UK); Lt Col Matthew Wordsworth (Imperial College NHS Trust, London, UK); Col Alistair Mountain (University Hospitals of North Midlands, Stoke on Trent, UK); Lt Col Rachel Howes (Oxford University Hospitals NHS Trust, Oxford, UK); Col Paul Parker, Gp Capt Ian Sargeant, Col Scott Adams (Royal Centre for Defence Medicine, Birmingham, UK); Col Hugo Guthrie (St George’s University Hospitals NHS Foundation Trust, London, UK); Surg Cdr Chris Hand (22International Committee of Red Cross, London, UK); Lt Col Guang Yim (The Welsh Centre for Burns and Plastic Surgery, Swansea, UK).

## Supplementary Material

zraf173_Supplementary_Data

## Data Availability

All data generated or analysed during this study are included in this published article (and its *supplementary information files*).
