# Correction to: Clinical guidelines for complex extremity war wound management: update and consensus using a mixed-method approach

**DOI:** 10.1093/bjsopen/zrag029

**Published:** 2026-03-18

This is a correction to: Samuel Snelling, Henry Claireaux, Harrison Roocroft, Kyung-Hoon Moon, Johann Jeevaratnam, Neil Eisenstein, Robert M T Staruch, Clinical guidelines for complex extremity war wound management: update and consensus using a mixed-method approach, *BJS Open*, Volume 10, Issue 1, February 2026, zraf173, https://doi.org/10.1093/bjsopen/zraf173

The names of the first two authors have been emended to read: “Samuel Snelling, Henry Claireaux”.

