# Supplementary material for: Clinical guidelines for complex extremity war wound management: update and consensus using a mixed-method approach
Source: BJS Open. 2026 Mar 5;10(1):zraf173. doi: 10.1093/bjsopen/zraf173 (PMC12961382; doi:10.1093/bjsopen/zraf173)
Supplement: zraf173_Supplementary_Data [file zraf173_supplementary_data.zip › Supplementary_material.docx]

**Title: Clinical Guidelines for Complex Extremity War Wound Management: Update and Consensus Using a Mixed-Method Approach**

Authors

Snelling S^1^, Claireaux H^2-4^, Roocroft H^1^, Moon K^2,5^, Lower Limb Debridement for Operations Working Group, Jeevaratnam J^6^, Eisenstein N^2^, Staruch RMT^1,7^

On behalf of the Lower Limb Debridement for Operations Working Group

1. Academic Department of Military Surgery & Trauma, UK
2. Academic Department of Military Trauma & Orthopaedics, UK
3. Department of Materials, Imperial College London, South Kensington Campus, London, SW7 2AZ, UK
4. Centre for Bacterial Resistance Biology, Imperial College London, South Kensington Campus, London, SW7 2AY, UK
5. Centre for Injury Studies, Imperial College, White City Campus, London, W12 7TA
6. Joint Hospital Group, Defence Medical Services, UK
7. Department of Plastic & Reconstructive Surgery, Oxford University Hospitals NHS Trust, Oxford, OX3 7LE

**Corresponding author.** Corresponding Author:

Robert MT Staruch,

[robmtstaruch@gmail.com](mailto:robmtstaruch@gmail.com).

Dove House Farm, OX44 9HG

Orchid ID: https://orcid.org/0000-0002-9114-7029

**Supplementary Materials – Index – Nil Extra**

| **References** | *Page 15 - 18* |
| --- | --- |
|  |  |

**Supplementary Methods**

**Supplementary Results**

**Supplementary Appendixes**

**Supplementary Figures and Tables**

**References**
